# Molecular Regulation of Carotenoid Accumulation Enhanced by Oxidative Stress in the Food Industrial Strain *Blakeslea trispora*

**DOI:** 10.3390/foods14091452

**Published:** 2025-04-23

**Authors:** Jiawei Deng, Yuyang Chen, Siting Lin, Yilu Shao, Yuan Zou, Qianwang Zheng, Liqiong Guo, Junfang Lin, Moutong Chen, Zhiwei Ye

**Affiliations:** 1 College of Food Science, South China Agricultural University, Guangzhou 510642, China; 2 Research Center for Micro-Ecological Agent Engineering and Technology of Guangdong Province, Guangzhou 510642, China; 3 Guangdong Provincial Key Laboratory of Microbial Safety and Health, State Key Laboratory of Applied Microbiology Southern China, Institute of Microbiology, Guangdong Academy of Sciences, Guangzhou 510070, China

**Keywords:** *Blakeslea trispora*, oxidative stress, carotenoids, biosynthesis, heat shock protein

## Abstract

*Blakeslea trispora* is a key industrial strain for carotenoid production due to its rapid growth, ease of cultivation, and high yield. This study examined the effects of oxidative stress induced by rose bengal (RB) and hydrogen peroxide (H_2_O_2_) on carotenoid accumulation, achieving maximum yields of 459.38 ± 77.15 μg/g dry cell weight (DCW) at 0.4 g/L RB and 294.38 ± 14.16 μg/g DCW at 0.6% H_2_O_2_. These results demonstrate that oxidative stress promotes carotenoid accumulation in *B. trispora*. To investigate the underlying molecular mechanisms, transcriptional levels of key genes were analyzed under optimal stress conditions. In the carotenogenic pathway, only *HMGR* showed upregulation, while *ACC*, linked to fatty acid biosynthesis, remained unchanged. Within the mitogen-activated protein kinase (MAPK) pathway, *FUS3* transcription increased under both stress conditions, *MPK1* transcription rose only under H_2_O_2_ stress, and *HOG1* exhibited no significant changes. Among heat shock proteins (HSPs), only *HSP70* showed elevated transcription under H_2_O_2_ stress, while other *HSP* genes remained unchanged. These findings suggest that oxidative stress induced by RB and H_2_O_2_ enhances carotenoid accumulation in *B. trispora* through distinct regulatory pathways. This study provides valuable insights into stress-adaptive mechanisms and offers strategies to optimize carotenoid production in fungi.

## 1. Introduction

Carotenoids, widely distributed in nature and representing one of the major classes of natural pigments [1,2], are extensively used in the food industry as natural colorants, imparting vibrant yellow to red hues to products such as beverages, dairy items, and baked goods. In photosynthetic organisms, carotenoids are integral components of the photosynthetic machinery and help mitigate oxidative stress and excessive radiation [3,4]. Carotenoids also offer numerous health benefits and hold significant potential for applications in medicine, food, and healthcare. Their prominent functional properties include lowering oxidative stress, regulating lipid metabolism, protecting vision, slowing the progression of neurodegenerative diseases, safeguarding the cardiovascular system, inhibiting cancer cell growth and proliferation, and improving immunological function [5,6]. The primary sources of carotenoids include plants, microalgae, and fungi, with production methods involving plant extraction, chemical synthesis, and microbial fermentation [7,8]. Among these, the microbial fermentation method has gained significant attention worldwide due to its independence from environmental factors, and the resulting products are considered safer and more cost-effective. Introducing stress factors in microbial fermentation has been demonstrated to significantly enhance carotenoid production. For example, oxidative stress induced by 0.4 g/L KMnO_4_ leads to the maximum carotenoid accumulation in the edible fungus *Cordyceps militaris* [9]. Similarly, subjecting red yeast *Rhodotorula glutinis* to 10 mM hydrogen peroxide (H_2_O_2_) stress significantly increased its carotenoid content [10]. In addition, the photosensitizer rose bengal (RB), which primarily generates ^1^O_2_ upon excitation by visible light, has been shown to induce carotenoid accumulation in *Phaffia rhodozyma* and *Chlamydomonas reinhardtii* [11,12]. As a result, H_2_O_2_ and RB were selected as oxidative stress factors to investigate carotenoid accumulation in *Blakeslea trispora* in this study.

*B. trispora* is a well-known strain for β-carotene production, employing co-culture fermentation with (+) and (−) mating types of *B. trispora* to produce rich triphosphate, which in turn stimulates β-carotene biosynthesis. The advantages of *B. trispora* for β-carotene biosynthesis include its rapid growth, ease of cultivation, and high yield per unit biomass. To enhance carotenoid production in *B. trispora*, previous studies have demonstrated that supplementing with glycerol can boost biosynthesis by more than tenfold, while improving oxygen flux can elevate production by up to fourfold [13,14]. Additionally, light and oxidative stresses play crucial roles in carotenoid biosynthesis in *B. trispora*, with a notable interaction between these factors. Blue light and reactive oxygen species (ROS) have been shown to influence the transcription and translation of carotenogenic genes, thereby promoting carotenoid accumulation in *B. trispora* [15].

The metabolism of carotenoids is influenced by various factors, including light, temperature, hormones, nutrients, and developmental signals [16]. Carotenoids, as secondary metabolites, play a role in adaptive responses to certain environmental stresses, including oxidative and osmotic stress [17]. In addition to carotenogenic genes, the biosynthesis and accumulation of carotenoids are also related to the metabolism of fatty acids. Acetyl-coenzyme A (acetyl-CoA), the initial substrate for isopentenyl pyrophosphate (IPP) biosynthesis, can also participate in other metabolic pathways, such as fatty acid biosynthesis in *Rhodosporidium toruloides* [18]. Carotenoid accumulation is closely related to fatty acid production, especially to unsaturated fatty acids (UFAs) like oleic acid, which enhance membrane fluidity and promote liposome formation [19,20]. The mitogen-activated protein kinase (MAPK) pathway regulates a range of cellular responses, including differentiation, proliferation, migration, and apoptosis. This signaling pathway transmits external signals to the nucleus through a series of phosphorylation events, impacting gene expression and cellular functions [21]. The MAPK pathway is an evolutionarily conserved signaling cascade that is widely present in eukaryotes. It involves upstream activators known as MAPK kinase kinases (MAPKKKs), which activate MAPK kinases (MAPKKs) [22]. These MAPKKs in turn phosphorylate a variety of effector proteins and transcription factors, thereby triggering diverse cellular and stress responses [23]. The five main MAPK pathways include FUS3 (mating), KSS1 (filamentous growth), HOG1 (response to high osmotic stress response), SLT2 (cell wall integrity), and SMK (ascospore formation) [24]. For instance, the fungus *Candida albicans* relies on the HOG1 MAPK pathway to adapt to osmotic stress during host invasion, with the significant upregulation of *HOG1* observed under high-osmotic conditions [25]. In contrast, in *C. militaris*, NaCl-induced stress leads to the downregulation of *FUS3* transcription while upregulating the transcription of the gene *ACC*, which encodes acetyl-CoA carboxylase, thereby inhibiting mycelial development. Meanwhile, oxidative stress induced by KMnO_4_ increases the transcription levels of *FUS3*, *HOG1*, *PSY*, and *HSP70*, indicating distinct regulatory responses to osmotic and oxidative stress [9].

Heat shock proteins (HSPs) are a class of molecular chaperones that are highly expressed under stress conditions, such as heat shock and oxidative stress. Under these conditions, proteins are prone to denaturation [26], and HSPs play a protective role by assisting in proper protein folding and preventing aggregation. These proteins are conserved across all domains of life, including plants, fungi, bacteria, and mammals, and are essential for processes like protein folding, transport, and degradation, providing essential protection during environmental stress. Based on their molecular weight, HSPs are categorized into families such as small HSPs, HSP20, HSP40, HSP60, HSP70, HSP90, and DanJ [27]. The core regulatory factor of the heat shock response (HSR) is the heat shock transcription factor (HSF), an evolutionarily conserved transcription factor in eukaryotes. The HSF is the primary regulator of the HSR, rapidly activating HSP expression upon heat stress. As molecular chaperones, HSPs safeguard cellular components by preventing protein denaturation and aggregation, thereby maintaining cellular integrity under stress conditions [28].

As previously described, oxidative stress plays a significant role in the biosynthesis and accumulation of carotenoids in fungi [15,29]. This study investigated the effects of abiotic stress induced by both RB and H_2_O_2_ on the hyphal morphology of *B. trispora* and the corresponding carotenoid biosynthesis and accumulation. Subsequently, quantitative real-time PCR (qRT-PCR) was employed to analyze the transcriptional levels of genes associated with carotenoid biosynthesis and related pathways in *B. trispora* under the optimal stress concentrations of RB and H_2_O_2_. The genes analyzed included *CarB*, *CarG*, *CarRA*, and *HMGR* in the carotenoid synthesis pathway. The analyzed genes included *CarB*, *CarG*, *CarRA*, and *HMGR* from the carotenoid biosynthesis pathway; *FUS3*, *HOG1*, and *MPK1* from the MAPK pathway; *ACC* from the fatty acid metabolic pathway; and *HSP20*, *HSP70*, *HSP100*, and *DanJ* from the heat shock protein family. The primary purpose of this study was to elucidate the regulatory mechanisms of key pathways in carotenoid biosynthesis in *B. trispora* under oxidative stress, focusing on the constraints and interactions among these pathways. These findings provide insights into the production of fungal secondary metabolites under abiotic stress and suggest new strategies for advancing carotenoid biosynthesis and accumulation research.

## 2. Materials and Methods

### 2.1. Fungal Strain

The strains used in this study were *B. trispora* mating types (+) (CCTCC AF 97006) and (−) (CCTCC AF 96002), which, preserved in activated cultures, were obtained from the Typical Culture Collection Centre (Wuhan, China).

### 2.2. Culture and Growth Analysis of B. trispora

The *B. trispora* (+) and (−) strains were activated separately by inoculating mycelium preserved on slant agar onto sterilized Potato Dextrose Agar (PDA) medium (200 g/L potato, 20 g/L glucose, 3 g/L KH_2_PO_4_, 1.5 g/L MgSO_4_, 20 g/L agar). Cultures were then incubated by being inverted in a biochemical incubator (LRH-250, Yiheng, Shanghai, China) at 28 °C in the dark for three days. Subsequently, a puncher (Φ = 6 mm) was used to perforate the activated culture, and three pieces of the activated culture were transferred into a 250 mL flask containing 100 mL of seed liquid culture medium (10 g/L glucose, 5 g/L yeast extract, 0.9 g/L K_2_HPO_4_, 4 g/L KH_2_PO_4_, 0.25 g/L MgSO_4_·7H_2_O, 1 g/L NH_4_Cl, pH 5.3). The seed liquid culture was incubated on a shaker (MQT-60G, Minquan, Shanghai, China) at 28 °C and 200 rpm in the dark for two days. For fermentation, the activated seed culture of *B. trispora* was inoculated into the fermentation medium (10 g/L lactose, 10 g/L peptone, 0.9 g/L K_2_HPO_4_, 4g/L KH_2_PO_4_, 0.25g/L MgSO_4_·7H_2_O, 1 g/L NH_4_Cl, pH 5.3) at a 10% seeding ratio, with a (+) to (−) stain ratio of 1:4. Cultures were incubated at 28 °C and 200 rpm in the dark for six days. After fermentation, the broth was filtered through gauze to separate the biomass, which was subsequently dried at 60 °C to a constant weight [13,30].

### 2.3. Morphological Observation of Mycelium Under Stress Conditions

Activated *B. trispora* (+) and (−) strains grown on PDA medium were cut into 6 mm diameter pieces using a sterilized puncher. Three punched fungal pieces were transferred into 90 mL of fermentation medium in a 250 mL flask. The cultures were incubated at 28 °C in darkness on a shaker at 200 rpm for five days. Subsequently, stress factors (RB and H_2_O_2_) were added, and incubation continued for an additional day. Under the treatment of 0.4 g/L RB and 0.6% H_2_O_2_, *B. trispora* showed the highest accumulation of carotenoids. The hyphal morphology of these two groups was examined using an optical microscope (CKX53, Olympus, Tokyo, Japan) at 400× magnification. In this study, the RB, H_2_O_2_, and CK groups represent treatments with 0.4 g/L RB, 0.6% H_2_O_2_, and the control (without stress factors), respectively.

### 2.4. Determination of Carotenoids

After six days of fermentation, the culture was harvested and filtered through four layers of gauze, followed by filtration through filter paper. The biomass was then dried in an oven at 60 °C, accurately weighed, and ground into a fine powder using quartz sand. To this powder, 15 mL of petroleum ether (Fuyu Fine Chemical Co., Tianjin, China) was added, and the mixture was shaken at 37 °C and 200 rpm for one hour. After standing for 20 min, the carotenoid-containing supernatant was collected, and the extraction process was repeated once. The absorbance of the carotenoid extract was measured at 450 nm using a UV–visible spectrophotometer (Tianmei, Shanghai, China), with petroleum ether serving as the control. The carotenoid content (μg/g) was calculated using the following equation: Carotenoid content (μg/g) = (A × V × D)/(0.16 × W), where A is the absorbance value of the diluted sample at 450 nm, V is the volume of the extractant (petroleum ether) (mL), D is the dilution factor, 0.16 is the extinction coefficient of carotenoids, and W is the dry mass of the sample (g) [9].

### 2.5. Gene Expression Analysis

Total RNA was extracted from the hyphae of *B. trispora* in the 0.4 g/L RB treatment group, the 0.6% H_2_O_2_ treatment group, and the control group using the fungal RNA kit (Omega, Stamford, CT, USA), according to the manufacturer’s instructions. The integrity and concentration of the nucleic acids were assessed as previously described [31]. The RNA extracted showed a purity ratio of 1.93 at OD_260/280_, indicating high-quality RNA suitable for downstream applications. Template cDNA was synthesized from total RNA using TransScript One-Step *g*DNA Removal and cDNA Synthesis SuperMix (TransGen Biotech, Beijing, China) following the manufacturer’s protocol. In this study, the elongation factor 1-alpha gene (*Tef1*) (GenBank accession no. DQ070019.1) was selected as the internal control gene [32], and the primers used for qRT-PCR are provided in Table 1. qRT-PCR reactions were performed on a CFX96 Touch^TM^ Real-Time PCR detection System (Bio-Rad, Hercules, CA, USA) using PerfectStart Green qPCR SuperMix (TransGen Biotech, Beijing, China). Relative gene expression levels were calculated using the 2^−ΔΔCT^ method [33].

### 2.6. Statistical Analysis

Carotenoid content and qRT-PCR data were obtained from three independent experiments. Statistical significance was assessed using one-way analysis of variance (ANOVA) in SPSS 22.0 software (SPSS Inc., Chicago, IL, USA), with a significance level set at *p* < 0.05. Duncan’s multiple range test was employed to determine significant differences among the groups. Graphics were created using Graphpad Prism 8.0.2 (Graphpad Software Inc., San Diego, CA, USA).

## 3. Results and Discussion

### 3.1. Carotenoid Content of B. trispora Under Different Stress Conditions

**Figure 1:**
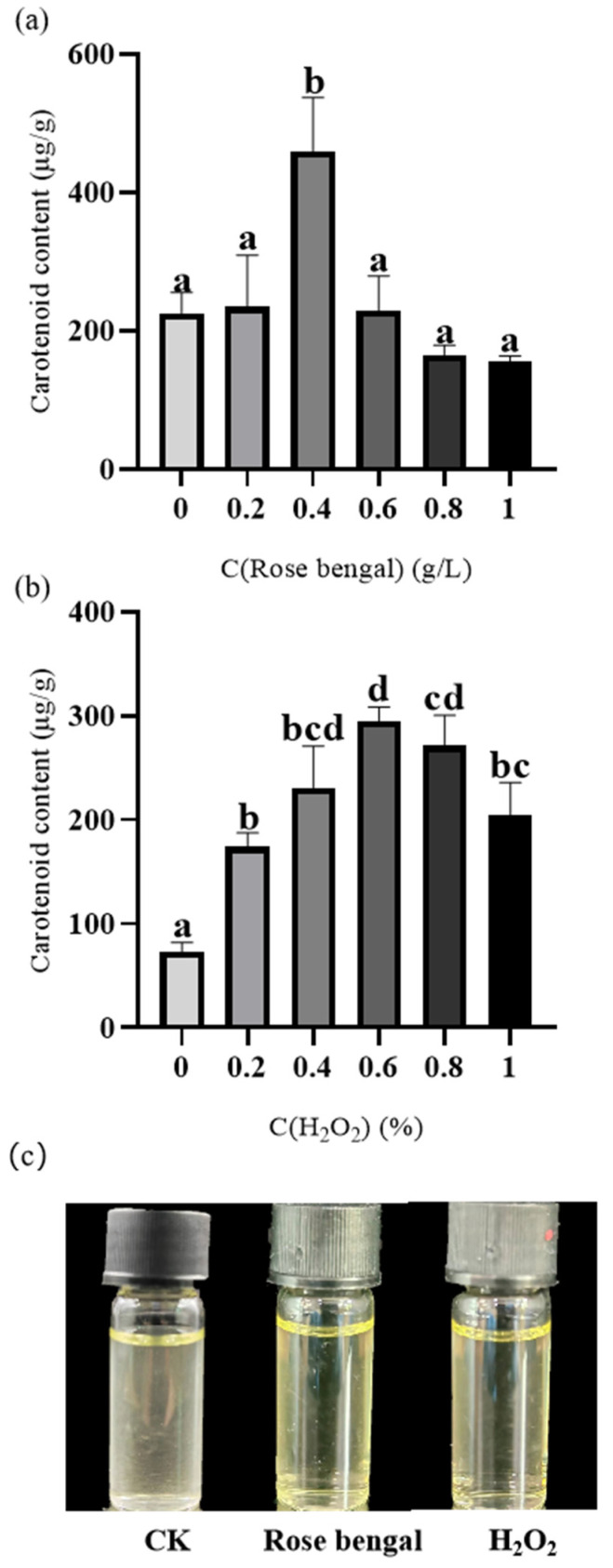
Carotenoid accumulation in *B. trispora* under stress conditions. (**a**) Rose bengal (RB) group; (**b**) H_2_O_2_ group; (**c**) carotenoid extract. The data indicated that carotenoid accumulation initially increased with a rising stress factor concentration but subsequently declined. Under H_2_O_2_ stress, carotenoid accumulation remained consistently higher than in the control group, whereas under RB stress, the two highest stress concentrations resulted in lower carotenoid accumulation compared to the control. This suggests that exceeding the optimal stress concentration inhibits carotenoid biosynthesis. The highest carotenoid production was observed under 0.4 g/L RB and 0.6% H_2_O_2_ stress, with yields of 459.375 ± 77.15 µg/g DCW and 294.375 ± 14.16 µg/g DCW, respectively, both significantly higher than the control group (*p* < 0.05). The lowercase letters in figures (**a**) and (**b**) represent significant differences between treatments.

Carotenoids were extracted from cultured *B. trispora* mycelium (Figure 1). As the concentration of stress factors increased, the carotenoid content exhibited an initial increase followed by a decline (Figure 1). Under RB stress, the carotenoid content increased steadily, peaking at 0.4 g/L, but it decreased at concentrations of 0.6 g/L to 1 g/L. Notably, the carotenoid levels were lower than those of the CK group at RB concentrations exceeding 0.8 g/L. Similarly, under H_2_O_2_ stress, the carotenoid content was higher than that in the CK group when the stress factor concentration ranged from 0.2% to 1%. Specifically, the carotenoid content increased continuously within the 0–0.6% concentration range, reaching a peak at 0.6%, before declining at concentrations between 0.8% and 1%. When the RB concentration was 0.4 g/L, the carotenoid content peaked at 459.38 ± 77.15 µg/g DCW, representing 2.04 times that of the control group. Similarly, at a H_2_O_2_ concentration of 0.6%, the carotenoid content reached its maximum of 294.38 ± 14.16 µg/g DCW, which is 4.03 times that of the control group (Figure 1).

### 3.2. Changes in Mycelial Morphology Under Stress

**Figure 2:**
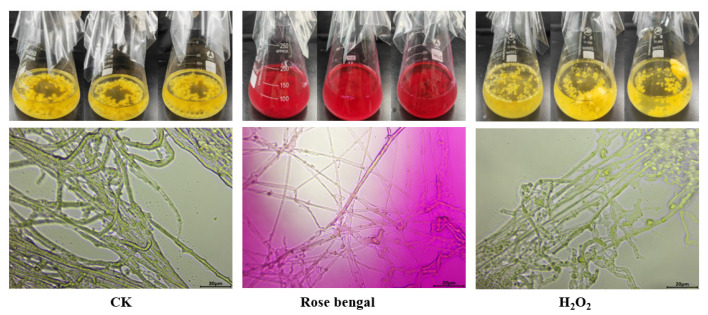
Morphological comparison of *B. trispora* under stress conditions. The images above show *B. trispora* mycelial growth under different conditions. Under the control (CK) condition, the mycelium grew uniformly, appeared lighter in color, and exhibited a healthy morphology. Under rose bengal (RB) stress, mycelial growth was significantly inhibited, the color darkened further, and mycelial aggregation was observed. Under H_2_O_2_ stress, mycelial growth was slowed, with some mycelia appearing broken or bent, and the color darkened, indicating signs of damage. The lower images, captured under a light microscope, show morphological differences among the groups. In the control group, the mycelium was relatively dense and thick. In the RB group, the mycelium was more dispersed and elongated. In the H_2_O_2_ group, the mycelium was sparser and more elongated. It is suggested that oxidative stress alters mycelial structure, potentially impacting carotenoid biosynthesis.

The morphology of mycelia was observed at 400× magnification using an optical microscope (Figure 2). *B. trispora*, a filamentous fungus, exhibited significant structural changes under different stress conditions. In the control group, the mycelia appeared denser and thicker, indicative of robust growth in the absence of oxidative stress. RB-induced oxidative stress resulted in mycelia that were more dispersed and thinner, whereas under H_2_O_2_-induced oxidative stress, the mycelium became more dispersed and relatively elongated, highlighting different morphological responses to the two stress conditions (Figure 2). Under oxidative stress conditions, fungal hyphae adapt by forming a denser and more highly branched network, increasing their absorptive surface area. This morphological adjustment enhances nutrient uptake efficiency, thereby sustaining the supply of terpenoid precursors essential for carotenoid biosynthesis and maintaining metabolic flux through carotenogenic pathways. Simultaneously, hyphae may differentiate into stress-resistant structures such as chlamydospores or sporangiophores. During this process, cells undergo vacuole partitioning and membrane lipid remodeling to form metabolically specialized compartments. These structural modifications enable the localized accumulation of carotenoids in specific cellular regions, such as spore walls or membrane domains, where they act as protective barriers against ultraviolet radiation and oxidative damage [34].

### 3.3. Expression of Genes Associated with Carotenoid Accumulation

**Figure 3:**
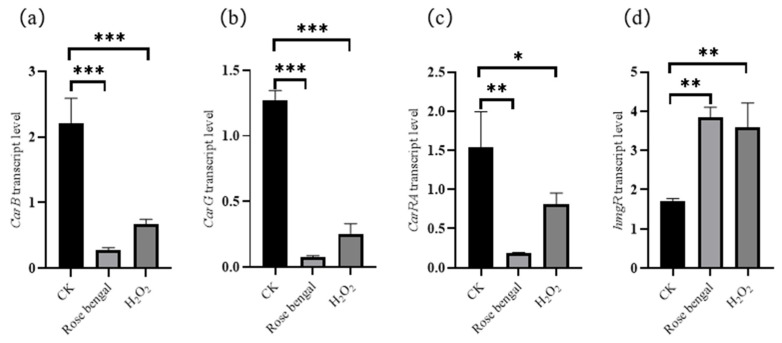
The transcription levels of carotenogenic genes of *B. trispora* under stress conditions. (**a**) Gene for phytoene dehydrogenase (*CarB*); (**b**) gene for geranylgeranyl pyrophosphate synthase (*CarG*); (**c**) gene for lycopene cyclase (*CarRA*); and (**d**) gene for 3-hydroxy-3-methylglutaryl-coenzyme A reductase (*HMGR*). The transcription level of *HMGR* was significantly higher in both the RB and H_2_O_2_ groups compared to the CK group, with a 2.26-fold and 2.10-fold increase, respectively. This suggests that *HMGR* plays a crucial role in carotenoid biosynthesis under oxidative stress. In contrast, the transcription levels of *CarB*, *CarG*, and *CarRA* were not significantly upregulated in the CK group and were notably downregulated under oxidative stress. These findings indicated that although *CarB*, *CarG*, and *CarRA* were essential in the carotenoid biosynthesis pathway, their expression may be negatively regulated or subject to negative feedback mechanisms in response to oxidative stress. Previous studies have shown that IPP, catalyzed by HMGR, is a key precursor of carotenoids, which are powerful antioxidants that can scavenge ROS (such as lutein and β-carotene). Under oxidative stress, the upregulation of *HMGR* activity may alleviate oxidative damage by enhancing carotenoid production [36]. Collectively, these findings suggest that HMGR may serve as a key regulatory point in the carotenoid biosynthesis pathway under oxidative stress conditions of rose bengal (RB) and H_2_O_2_ (*** *p* < 0.001, ** *p* < 0.01, * *p* < 0.05).

Carotenoids are essential secondary metabolites in *B. trispora*, and their content can significantly increase under optimal stress conditions. Based on previous research and data from the National Center for Biotechnology Information (NCBI, https://www.ncbi.nlm.nih.gov/), it is speculated that the expression products of twelve specific genes may contribute to carotenoid accumulation under stress conditions [23,24,35]. These include genes responsible for carotenogenic enzymes in the biosynthetic pathway, such as 3-hydroxy-3-methylglutaryl-CoA reductase (HMGR), phytoene dehydrogenase (CarB), geranylgeranyl pyrophosphate synthase (CarG), and bifunctional lycopene cyclase/phytoene synthase (CarRA). Additionally, regulatory genes in the MAPK pathway (*FUS3*, *HOG1*, and *MPK1*), along with genes coding for heat shock proteins (*HSP20*, *HSP70*, *HSP100*, and *DanJ*) and acetyl-CoA carboxylase (*ACC*) within the triglyceride metabolism pathway, may also contribute to the regulatory mechanisms underlying carotenoid biosynthesis and accumulation in *B. trispora* under stress conditions. As a result, the transcriptional levels of these four genes were analyzed in *B. trispora* mycelium samples under three conditions: the control group (CK), the RB treatment group (0.4 g/L RB), and the H_2_O_2_ treatment group (0.6% H_2_O_2_), using qRT-PCR analysis. The transcription levels of carotenoid biosynthesis pathway genes under stress conditions are shown in Figure 3. The transcription level of the *HMGR* gene was significantly higher in the RB and H_2_O_2_ treatment groups compared to the CK group, with an increase of 2.26 times in the RB group and 2.10 times in the H_2_O_2_ group. In contrast, the transcription levels of the *CarB*, *CarG*, and *CarRA* genes were significantly decreased in both the RB and H_2_O_2_ groups compared to the CK group. Specifically, in the RB group, the *CarB*, *CarG*, and *CarRA* transcription levels decreased by 7.93, 16.53, and 8.36 times, respectively, compared with the CK group. Similarly, in the H_2_O_2_ group, the transcription levels of these genes decreased by 3.29, 5.01, and 1.91 times, respectively, compared with the CK group.

**Figure 4:**
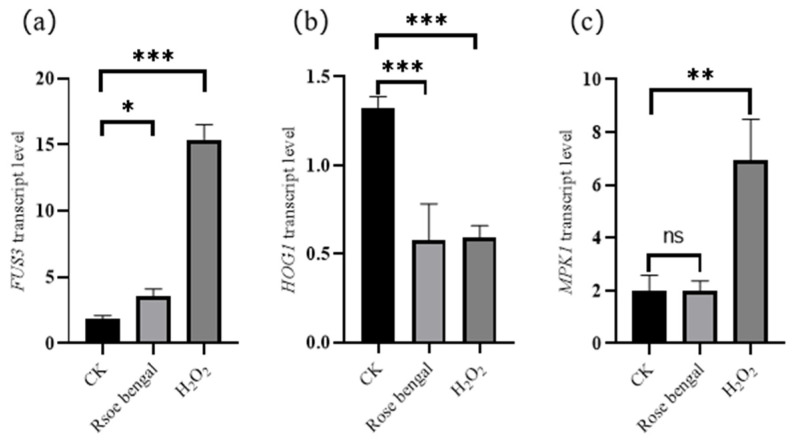
The transcription levels of genes involved in the mitogen-activated protein kinase (MAPK) pathway under stress conditions. (**a**) *FUS3*; (**b**) *HOG1*; (**c**) *MPK1*. The transcription levels of *FUS3* and *MPK1* were significantly upregulated in the H_2_O_2_ group, with *FUS3* increasing by 8.45 times and *MPK1* by 3.49 times, suggesting their involvement in the oxidative stress response. The transcription level of *HOG1* did not increase under either stress condition, indicating that oxidative stress might suppress its activity. As a result, the increased transcription of *FUS3* may promote mycelial growth and mating, subsequently enhancing carotenoid biosynthesis in *B. trispora* [9]. Under oxidative stress, *MPK1* may influence transcription factors that regulate carotenoid biosynthesis, thereby enhancing carotenoid production. The suppression of *HOG1* under oxidative stress suggests that a negative feedback regulation mechanism may contribute to increased carotenoid accumulation. It is indicated that the MAPK pathway plays a crucial role in regulating mycelial growth, stress response, and carotenoid biosynthesis in *B. trispora* under oxidative stress (*** *p* < 0.001, ** *p* < 0.01, * *p* < 0.05).

The transcription levels of the MAPK pathway genes *FUS3*, *HOG1*, and *MPK1* under RB and H_2_O_2_ stress conditions are presented in Figure 4. The transcription levels of *FUS3* and *MPK1* in the H_2_O_2_ group are significantly higher than those in the CK group, with *FUS3* and *MPK1* being 8.45 times and 3.49 times higher, respectively. The transcription level of *FUS3* in the RB group was also significantly increased compared to the CK group, increasing by 1.96 times. However, the transcription level of *MPK1* in the RB group decreased compared to the CK group, with a 0.99 reduction. Meanwhile, the transcription level of *HOG1* did not increase in either the RB or H_2_O_2_ treatment groups; instead, it was significantly downregulated by 2.29-fold and 2.22-fold, respectively, compared to the CK group.

**Figure 5:**
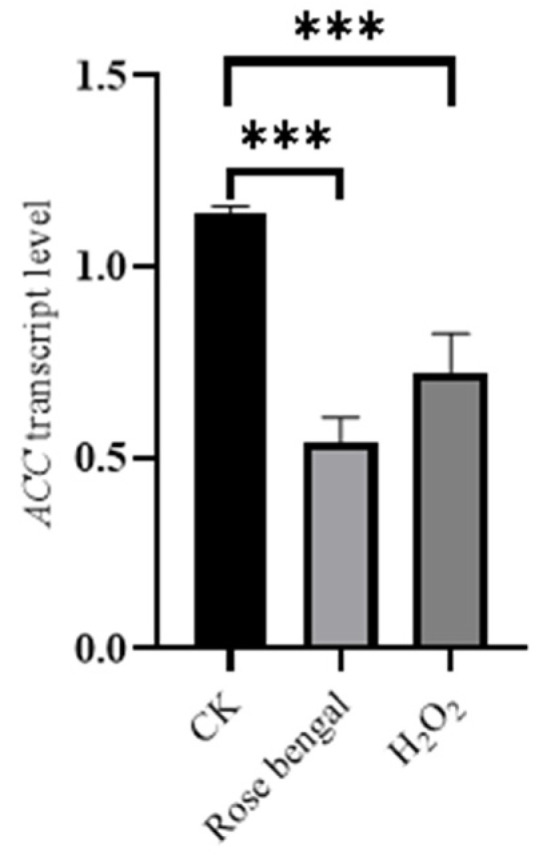
The transcription level of the gene for acetyl-CoA carboxylase (*ACC*) of the fatty acid biosynthetic pathway under stress conditions. As known, acetyl-coenzyme A (CoA) participates in carotenoid biosynthesis by serving as a precursor for isopentenyl pyrophosphate (IPP) through its conversion to 3-hydroxy-3-methylglutaryl coenzyme A (HMG-CoA) in the mevalonate (MVA) pathway. Additionally, acetyl-CoA is involved in several other essential metabolic pathways, including the tricarboxylic acid (TCA) cycle, fatty acid synthesis, and cholesterol synthesis, which provide energy and a favorable metabolic environment for carotenoid biosynthesis. In this study, under RB and H_2_O_2_ stress, the transcription level of *ACC* did not increase compared to the CK group. This suggests that acetyl-CoA may be allocated to other metabolic processes to regulate carotenoid biosynthesis and accumulation in *B. trispora* under oxidative stress conditions (*** *p* < 0.001).

**Figure 6:**
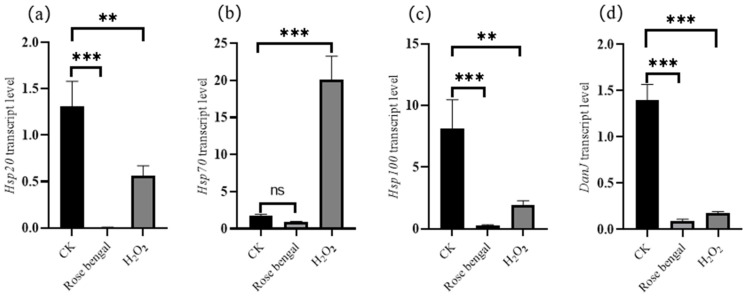
Effects of oxidative stress conditions on the transcription levels of heat shock proteins (HPSs). (**a**) *HSP20*, (**b**) *HSP70*, (**c**) *HSP100*, and (**d**) *DanJ*. Under RB and H_2_O_2_ stress, compared with the CK group, only *HSP70* in H_2_O_2_ showed a significant increase in transcription level (11.80-fold increase), while the RB group did not exhibit any increase in transcription level. This indicates that HSP70 plays a crucial role in carotenoid biosynthesis, potentially by stabilizing and prolonging the activity of carotenogenic enzymes, thereby enhancing carotenoid biosynthesis under oxidative stress. In contrast, the transcription levels of *HSP20*, *HSP100*, and *DanJ* did not increase in either stress condition, suggesting that these *HSPs* may not be directly involved in carotenoid biosynthesis in *B. trispora* under oxidative stress (*** *p* < 0.001, ** *p* < 0.01).

The transcription level of *ACC* in the fatty acid biosynthesis pathway under RB and H_2_O_2_ stress is shown in Figure 5. The transcription levels in both the RB and H_2_O_2_ groups, however, were significantly decreased compared to the CK group, with reductions of 2.11 times and 1.56 times, respectively. Meanwhile, the transcription levels of four genes, *HSP20*, *HSP70*, *HSP100*, and *DanJ*, in the HSP family under RB and H_2_O_2_ stress conditions are shown in Figure 6. The transcription levels of *HSP20*, *HSP100*, and *DanJ* were significantly decreased in both the RB and H_2_O_2_ groups compared to the CK group. Specifically, in the RB group, the transcription levels of *HSP70*, *HSP100*, and *DanJ* decreased by 210.34 times, 31.27 times, and 14.86 times, respectively, compared to the CK group. In the H_2_O_2_ group, the transcription levels of *HSP20*, *HSP100*, and *DanJ* decreased by 2.32 times, 4.23 times, and 7.94 times, respectively. Notably, *HSP70* showed divergent responses under the two stress conditions: it was significantly increased in the H_2_O_2_ group, with a transcription level 11.80 times higher than that in the CK group, but it decreased in the RB group by 1.78 times compared to the CK group.

## 4. Discussion

*B. trispora*, a member of the order Mucorales within the class Mucoromycetes, has attracted widespread attention for its potential to produce large quantities of carotenoids [13]. Carotenoids, essential secondary metabolites, exhibit diverse biological functions, including acting as antioxidants and playing roles in cellular protection mechanisms. This study investigates the effects of RB and H_2_O_2_ stress factors on carotenoid biosynthesis and accumulation in *B. trispora*. The findings reveal that these stressors significantly influence fungal growth, carotenoid production, and expression of genes associated with carotenogenesis in *B. trispora*.

Primary carotenoids are integral to the structural and functional components of the photosynthetic apparatus, whereas secondary carotenoids are biosynthesized in response to stress. These secondary carotenoids are stored in oil droplets, serving to form a protective layer under stress conditions and imparting the characteristic pink or red coloration observed in certain stress-tolerant algae [37]. The antioxidant properties of carotenoids mitigate damage caused by ROS, prevent lipid peroxidation, and promote the stability of microbial mechanisms [3,38]. This study investigates the effects of varying concentrations of RB and H_2_O_2_ stress on carotenoid production in *B. trispora*. The results indicate that the production of carotenoids was significantly enhanced under RB concentrations from 0.2 g/L to 0.6 g/L and H_2_O_2_ concentrations ranging from 0.2% to 1%. The optimal stress conditions for carotenoid accumulation were determined to be 0.4 g/L RB and 0.6% H_2_O_2_. Under these conditions, *B. trispora* exhibited the highest carotenoid production, reaching 459.375 ± 77.15 µg/g DCW and 294.375 ± 14.16 µg/g DCW, respectively (Figure 1). At lower levels, oxidative stress-induced ROS function as signaling molecules, triggering cellular antioxidant defense pathways. This activation upregulates the activity of key enzymes in the mevalonate (MVA) and 2-*C*-methyl-d-erythritol 4-phosphate (MEP) pathways, such as HMGR and 1-deoxy-d-xylulose-5-phosphate reductoisomerase, thereby enhancing the production of IPP, a crucial precursor for carotenoid biosynthesis. This, in turn, promotes the biosynthesis of downstream intermediates like geranylgeranyl pyrophosphate (GGPP). However, excessive oxidative stress can inactivate essential carotenogenic enzymes such as phytoene desaturase, ultimately leading to reduced carotenoid production. Notably, RB predominantly generates oxidative stress via ^1^O_2_ and a small amount of ·OH, whereas H_2_O_2_ induces stress mainly through ·OH and O_2_^−^. These ROS exert distinct oxidative effects on intracellular targets, reflecting their different molecular mechanisms and modes of action within cellular metabolic pathways [39,40].

Carotenoids are generally a general term for hydrocarbons and their derivatives, which are chemically composed of eight isoprene units [5]. To date, more than 750 different carotenoids have been identified, typically existing as stable all-trans isomers [2]. The precursors for carotenoid biosynthesis are generated via two primary pathways: the MVA pathway and the MEP pathway [8]. In plant plastids, IPP and its isomer dimethylallyl pyrophosphate (DMAPP) are biosynthesized through the MEP pathway, while in fungi, these intermediates are generated via the MVA pathway. Carotenoid biosynthesis can be divided into three major stages: (i) the generation of the precursors IPP and DMAPP, (ii) the formation of GGPP, and (iii) the production of carotenoids followed by their downstream modifications. In carotenoid-producing organisms, downstream modifications of the carotenoid backbone include desaturation, cyclization, and oxidation, along with further modifications such as glycosylation and oxidative cleavage [4,41]. In carotenoid biosynthesis, the precursor molecule IPP is generally converted to phytoene through the enzymatic actions of IPP isomerase, GGPP synthase (GGPS), and phytoene synthase (PSY). Phytoene, the first synthesized C_40_ carotenoid, subsequently undergoes a series of enzymatic reactions, including oxidation, hydrogenation, dehydrogenation, hydroxylation, cyclization, epoxidation, and carbon–nitrogen rearrangement, leading to the production of the diverse carotenoids found in nature [42,43]. In the carotenoid biosynthesis pathway of *B. trispora*, the activity of the bifunctional enzyme CarRA, which functions as both a lycopene cyclase and phytoene synthase, determines the level of lycopene accumulation, particularly through its cyclization ability. CarB primarily facilitates the conversion of phytoene into lycopene, working in conjunction with CarRA to regulate carotenoid biosynthesis. CarG is responsible for biosynthesizing GGPP, the immediate precursor of carotenoids. Additionally, HMGR is the first rate-limiting enzyme in the MVA pathway and serves as a critical regulatory site in terpenoid biosynthesis. The expression level of the *HMGR* gene is positively correlated with carotenoid biosynthesis in *B. trispora* [35]. In this study, the transcription levels of the genes involved in carotenoid biosynthesis showed a significant increase in *HMGR* under both RB and H_2_O_2_ groups, indicating that *HMGR* plays a direct role in carotenoid biosynthesis under these conditions. However, the transcription levels of *CarB*, *CarG*, and *CarRA* were either statistically unchanged or significantly decreased under RB and H_2_O_2_ stress. It is hypothesized that *B. trispora* may reduce the degradation (half-life) of carotenogenic enzymes associated with these three genes through the action of molecular chaperone HSP70, thereby maintaining carotenoid biosynthesis. This strategy would prioritize the preservation of enzyme function over the enhancement of certain carotenogenic gene transcription levels, allowing the organism to conserve substrates and energy while coping with stress conditions.

The MAPK cascade consists of a three-tiered protein kinase module, where MAPKKK phosphorylates MAPKK, which then activates a MAPK through the dual phosphorylation of a conserved threonine and tyrosine residue pair [44]. In *Saccharomyces cerevisiae*, at least five MAPK pathways have been identified: FUS3, KSS1, HOG1, SLT2, and SMK, each involved in different processes such as mating, filamentous growth, high osmolality response, cell wall integrity, and ascospore formation, respectively [24]. The effects of RB and H_2_O_2_ stress on the expression of three genes in the MAPK pathway (*FUS3*, *HOG1*, and *MPK1*) are shown in Figure 4. The transcription levels of *FUS3* and *MPK1* were significantly higher in the H_2_O_2_ group than in the CK group. In the RB group, *FUS3* expression was also significantly elevated compared to the CK group, whereas *MAP1* expression decreased. Additionally, *HOG1* transcription did not increase in either the RB or H_2_O_2_ groups but was significantly lower than in the CK group. It is speculated that the increased transcription of *FUS3* promotes mycelial growth and mating, which subsequently enhances carotenoid biosynthesis in *B. trispora.* Under oxidative stress, MAPK coded by gene *MKP1* may influence the transcription factors involved in regulating carotenoid biosynthesis, thereby affecting carotenoid accumulation. However, in this study, oxidative stress appears to inhibit the function of the HOG1 MAPK pathway. ROS, common byproducts in stress conditions, can activate the MAPK cascade through phosphorylation events. Activation of the MAPK cascade triggers internal antioxidant systems to scavenge ROS via enzymatic mechanisms, involving superoxide dismutase, catalase, glutathione peroxidase, and ascorbate peroxidase, as well as non-enzymatic mechanisms, where ROS are eliminated by internal antioxidants such as glutathione, α-tocopherol, and carotenoids [45]. In *Dunaliella salina*, ROS induced by oxidative stress can affect the cell growth process, the production of photosynthetic pigments, and secondary metabolites, including the biosynthesis and accumulation of carotenoids [46]. The MAPK pathway includes key genes such as *FUS3*, *HOG1*, *KSS1*, *SLT2*, *SMK*, and *MPK1*. The results of this study indicate that *FUS3* and *MPK1* primarily mediate carotenoid accumulation under RB stress, while *MPK1* is the main regulator of carotenoid biosynthesis under H_2_O_2_ stress.

The biosynthesis of carotenoids shares a common precursor, acetyl-CoA, with the biosynthesis of fatty acids. In the carotenoid biosynthesis pathway, acetyl-CoA is converted to IPP and DMAPP via the MVA pathway, serving as direct precursors for carotenoid biosynthesis. IPP and DMAPP subsequently condense to form phytoene, marking the initial step in carotenoid biosynthesis. Additionally, studies have found that excessive carotenoid production is associated with oil pellet formation and a decrease in the degree of unsaturated fatty acids [47]. As shown in Figure 5, the transcription level of the acetyl-CoA carboxylase gene, *ACC*, in the fatty acid biosynthesis pathway significantly decreased under RB and H_2_O_2_ stress compared to the control group. It is speculated that oxidative stress may stimulate the biosynthesis and accumulation of carotenoids as a defense mechanism against oxidative stress, leading to a reduction in fatty acid synthesis. As a result, the transcription level of *ACC* decreased.

HSPs are a family of conserved proteins that enhance stress resistance and protect cells from external damage [48]. During heat stress, HSFs, which are initially bound to *HSP70* and *HSP90*, are released. This allows chaperone proteins to associate with misfolded proteins, degrade abnormal proteins, repair protein misfolding, and maintain protein homeostasis within cells. Once released, HSFs bind to conserved sequences known as heat shock elements (HSEs) in the promoters of downstream response genes, activating their expression [49,50]. Post-translational modifications, such as phosphorylation, acetylation, and polymerization, can regulate HSF activity. However, due to the presence of large gene families for both HSFs and protein kinases, the phosphorylation of HSFs is quite complex [51,52]. In this study, the transcription levels of four HSP family genes (*HSP20*, *HSP70*, *HSP100*, and *DanJ*) under RB and H_2_O_2_ stress are shown in Figure 6. The transcription levels of *HSP20*, *HSP100*, and *DanJ* in the RB and H_2_O_2_ groups were significantly lower than those in the CK group. The decreased transcription levels may result from the prioritization of energy resources toward the critical pathways for resistance to oxidative stress, alongside the specific inhibition of the heat shock pathway under oxidative stress. Meanwhile, the carotenoids produced by RB-treated *B. trispora* may be sufficient to counteract ROS-induced damage [53,54]. Conversely, the transcription level of *HSP70* in the H_2_O_2_ group was significantly higher than in the CK group, while it was significantly lower in the RB group. It is speculated that HSP70, as a molecular chaperone, helps proteins to fold correctly under oxidative stress, maintaining protein homeostasis in cells and reducing unnecessary energy consumption. This process may help prolong the activity of carotenogenic enzymes in *B. trispora*, promoting carotenoid production to counteract oxidative stress.

## 5. Conclusions

Carotenoids, as bioactive pigments with antioxidant properties, are widely utilized in the food and nutraceutical industries for their natural coloring capacity and potential health benefit. This study demonstrated that oxidative stress significantly influences the biosynthesis and accumulation of carotenoids in *B. trispora*. The highest carotenoid accumulations of 459.375 ± 77.15 µg/g DCW and 294.375 ± 14.16 µg/g DCW were observed under oxidative stresses of 0.4 g/L RB and 0.6% H_2_O_2_, respectively. This study demonstrates that RB stress affects carotenoid biosynthesis in *B. trispora* primarily through the *HMGR* gene in the upstream carotenogenic pathway and *FUS3* in the *MAPK* pathway. In contrast, H_2_O_2_ influences this process mainly via *HMGR* in the carotenogenic pathway, *FUS3* and *MPK1* in the MAPK pathway, and *HSP70* from the heat shock protein family. Under oxidative stress (RB and H_2_O_2_), HMGR appears to be directly involved in carotenoid biosynthesis. The activation of the MAPK pathways, specifically FUS3 signaling, may help *B. trispora* resist oxidative conditions by regulating the related metabolic processes. In addition, the activation of the *MPK1* gene and the *MAPK* pathway in response to oxidative stress factors may control carotenoid production [55]. The transcription level of *HSP70* increased significantly under RB and H_2_O_2_ stress, suggesting that HSP70 may facilitate carotenoid accumulation by stabilizing carotenogenic enzymes, hereby enhancing the organism’s resistance to oxidative stress. As a result, by overexpressing highly transcribed genes identified under oxidative stress conditions, high-yield carotenoid-producing recombinant strains of *B. trispora* could be developed through targeted genetic engineering. This study lays a foundation for further elucidating carotenoid biosynthesis in *B. trispora* and supports the advancement of more efficient industrial production strategies, highlighting its practical significance in biotechnology and the food industry.

## Figures and Tables

**Table 1:** Oligonucleotide primer sequences were used in this study.

Gene	Description	GenBank Accession Number	Primer Sequence (Forward/Reverse)
*Tef1*	elongation factor 1-alpha	AF157235	AACTCGGTAAGGGTTCCTTCAAG CGGGAGCATCAATAACGGTAAC
*CarB*	phytoene dehydrogenase	AY176663	CGCTTGCACTTGTTTGGTAAGATC CAACCATGTTGAAACCACCACG
*CarG*	geranylgeranyl pyrophosphate synthase	JQ289995	CCCAAGGACGATTTGATCGTC CATGTTGGGCTTGTTCAACTTGG
*CarRA*	bifunctional lycopene cyclase/phytoene synthase	AY17666	CCCAACACTTCTTGGAAGCAAAC GCCACAGGTCGACGCAAGA
*HMGR*	3-hydroxy-3-methylglutaryl-coenzyme A reductase	KAI8378970	GATCTCCCGTGACTCTCGTAC CCTTCCGTAGTTGCCATGGG
*FUS3*	mitogen-activated protein kinase	JAIWNE010000030 BD560DRAFT_397017	CGCTGGTATCGTGCACCTG CATCCATTGTAGGTGTGCCGAG
*HOG1*	mitogen-activated protein kinase	JAIWNE010000026 BD560DRAFT_454271	GTGTCTACCAGATACTATCGTGC GCAATCAAATCATCGGATGGTG
*HSP20*	heat shock protein 20	JAIWNE010000004 BD560DRAFT_383523	CCAGCCACGGATATGATTGAG GTTTTTCATTCGCATCCTTAGGC
*HSP70*	heat shock protein 70	JAIWNE010000001 BD560DRAFT_380704	CTATGGCACTGTGATTGGTATCG CAGTACGTGTAGGGTTAGCAGAG
*HSP100*	heat shock protein 100	JAIWNE010000035 BD560DRAFT_398661	CCAACTTTTGCTTCAAGTCACGG GAACGTAAACGCTTCCAACTCG
*MPK1*	mitogen-activated protein kinase	JAIWNE010000044 BD560DRAFT_401363	CTTCACCTGAGGGTAATGCTGG CCTTTGAAGAGAGGTCGGCC
*DanJ*	DanJ domain protein	JAIWNE010000001 BD560DRAFT_380444	GCCTACGAAATTCTATCTGATCC GTAGGTCTGTCCATCATGAAAGGC
*ACC*	Acetyl-CoA carboxylase	JAIWNE010000022 BD560DRAFT_446170	CCCCAGACTTCAAGTTGAGCATC GTAGGACGACGATGGCTTGTC

## Data Availability

The original contributions presented in this study are included in the article. Further inquiries can be directed to the corresponding author.
